# Therapeutic Outcomes of Fingolimod and Interferon Beta-1a in Relapsing–Remitting Multiple Sclerosis: A Real-World Study from Jordan

**DOI:** 10.3390/medicina62010203

**Published:** 2026-01-18

**Authors:** Arwa Al Anber, Ola Abu Al Karsaneh, Dua Abuquteish, Osama Abdallah, Mohammad A. Issa, Mohammad Sa’adeh, Dena Kilani

**Affiliations:** 1 Department of Pharmacology and Public Health, Faculty of Medicine, The Hashemite University, Zarqa 13133, Jordan; 2 Department of Microbiology, Pathology and Forensic Medicine, Faculty of Medicine, The Hashemite University, Zarqa 13133, Jordan; olaa@hu.edu.jo (O.A.A.K.); dua@hu.edu.jo (D.A.); 3 Medical Intern, Ministry of Health, Amman 1118, Jordan; osama.abdallah42@gmail.com (O.A.); mohammadissa2000us@gmail.com (M.A.I.); mohammedamjad231@gmail.com (M.S.); dena.kilani1999@icloud.com (D.K.)

**Keywords:** multiple sclerosis, relapsing–remitting MS, fingolimod, interferon beta-1a, annualized relapse rate, Jordan

## Abstract

*Background and Objectives*: Multiple sclerosis (MS) is a chronic autoimmune disease of the central nervous system with rising prevalence in the Middle East. Real-world comparative data on disease-modifying therapies from this region remain limited. This retrospective study compared the clinical outcomes and tolerability of fingolimod and interferon beta-1a (IFN-β1a) among patients with relapsing–remitting multiple sclerosis treated at a large public referral hospital in Jordan. *Materials and Methods:* All eligible RRMS patients received fingolimod or IFN-β1a at a single tertiary hospital. The annualized relapse rate (ARR), Expanded Disability Status Scale (EDSS) scores, and adverse effect frequencies were analyzed using descriptive and inferential statistics. A full-cohort inclusion approach was applied instead of sample-size calculation, as all available cases at Al-Basheer Hospital (Amman, Jordan) were included. *Results:* Fingolimod-treated patients showed a significantly higher ARR than those on IFN-β1a (0.51 vs. 0.26, *p* = 0.016), an association likely influenced by treatment sequencing and baseline disease activity. EDSS distributions were similar between treatment groups, with most patients demonstrating mild disability (EDSS ≤ 3.5). IFN-β1a was linked to injection site reactions, while fingolimod was better tolerated. *Conclusions:* The higher observed relapse rate among fingolimod-treated patients possibly reflects treatment sequencing and underlying disease severity rather than pharmacologic efficacy, as fingolimod was commonly prescribed as an escalation therapy. These findings highlight the importance of individualized treatment selection and underscore the need for prospective studies incorporating standardized baseline disease activity measures to better inform multiple sclerosis care in Jordan and the wider Middle Eastern region.

## 1. Introduction

Multiple sclerosis (MS) is a chronic, immune-mediated demyelinating disorder of the central nervous system (CNS), marked by inflammatory lesions, axonal damage, and progressive neurological decline [1]. It ranks among the most common non-traumatic causes of disability in young adults and poses a substantial public health challenge worldwide [2]. Globally, the prevalence of MS continues to rise. According to the 2020 Atlas of MS, approximately 2.8 million people are living with the disease, with incidence rates and diagnostic capacity increasing worldwide [3]. In the Middle East, the pooled prevalence is estimated at 51.5 per 100,000, a figure that likely underrepresents the true burden due to variability in health system infrastructure and case ascertainment [4,5]. Jordan, specifically, has witnessed a steady rise in MS diagnoses, attributed to improved access to neuroimaging and specialist care, greater awareness, and possible changes in environmental risk factors [6,7].

MS typically presents between the ages of 20 and 40, with a pronounced female predominance (approximately two to three times more common in women than men) [8]. The disease course is highly variable and includes distinct phenotypes, including relapsing–remitting MS (RRMS), secondary progressive MS (SPMS), and primary progressive MS (PPMS). RRMS is the most prevalent form at diagnosis, characterized by episodic neurological symptoms followed by partial or complete recovery. However, over time, many RRMS patients transition into SPMS, where neurologic deterioration becomes more sustained and less responsive to therapy [9]. RRMS presents with episodic neurological dysfunction (such as optic neuritis, motor weakness, and sensory disturbances) reflecting multifocal CNS involvement. Diagnosis is established by demonstrating dissemination in time and space using clinical assessment and MRI-based McDonald criteria revisions, enabling earlier confirmation of RRMS [10,11].

Over the past two decades, the therapeutic landscape of MS has shifted considerably with the development of disease-modifying therapies (DMTs), aimed at reducing the frequency of relapses, delaying disability progression, and improving quality of life. Among the first approved agents was interferon beta-1a (IFN-β1a), which modulates immune activity by decreasing T-cell activation and cytokine expression while enhancing the integrity of the blood–brain barrier. IFN-β1a modestly reduces relapse rates and new lesion formation on MRI, but its injectable route and associated flu-like symptoms often affect adherence and patient satisfaction [12,13]. Fingolimod, approved by regulatory agencies in 2010, represents the first oral DMT for MS. It functions as a sphingosine-1-phosphate receptor modulator, effectively sequestering lymphocytes in lymph nodes and preventing their migration into the CNS. Clinical trials have demonstrated fingolimod’s superior efficacy in reducing relapse rates and MRI activity compared to placebo and IFN-β1a, though it carries a risk of adverse events such as bradycardia, macular edema, hepatotoxicity, and susceptibility to infections [14,15,16].

Despite the availability of randomized controlled trial evidence comparing fingolimod and IFN-β1a, real-world data from Middle Eastern populations remain limited, particularly within public healthcare systems where treatment availability, prescribing practices, and monitoring capacity may differ from those in trial settings. In Jordan, evidence derived from routine clinical practice is needed to better characterize treatment outcomes among patients with relapsing–remitting multiple sclerosis.

Accordingly, the objective of this study was to compare the real-world effectiveness and tolerability of fingolimod and IFN-β1a among patients with relapsing–remitting multiple sclerosis treated at a tertiary public hospital in Jordan. The primary outcome was the ARR. Secondary outcomes included disability status assessed using the EDSS and the frequency of treatment-related adverse effects.

## 2. Materials and Methods

### 2.1. Study Design and Setting

This retrospective, single-center cohort study included all RRMS patients treated with fingolimod or IFN-β1a at Al-Basheer Hospital, Amman, Jordan, which is the largest public referral hospital in the country. All eligible patients during the study period (January 2023–February 2024) were included to minimize selection bias. Formal a priori power or sample-size calculation was not performed because the study followed a retrospective, real-world, full-cohort design. All eligible patients treated with fingolimod or IFN-β1a at Al-Basheer Hospital during the study period were included, and the available patient population therefore defined the sample size. Such full-cohort inclusion approaches are common in real-world MS studies where the available patient population defines the sample size [17]. This strategy aimed to minimize selection bias and enhance the representativeness of treatment outcomes in routine clinical care. During the study period, IFN-β1a and fingolimod were among the few DMTs routinely available in Jordanian public hospitals. IFN-β1a remained widely used due to cost-effectiveness and availability.

Eligible participants were adults aged 18 years or older with a confirmed diagnosis of RRMS, established by a certified neurologist according to the 2017 revised McDonald criteria, at least 24 months prior to study entry. Inclusion required continuous treatment with either oral fingolimod or injectable IFN-β1a for a minimum of 24 months before enrollment. Patients diagnosed with progressive forms of MS were excluded as they were receiving other therapies.

### 2.2. Data Collection

Data were obtained through structured patient questionnaires and electronic medical records accessed via the national HAKEEM health information system [18]. Collected variables included demographic characteristics (age, sex, education, employment, marital status, smoking history), clinical features (disease duration, age at onset, initial presenting symptoms, current symptoms), treatment history (current and previous medications), EDSS scores, and treatment-related adverse effects, which were collected from medical records. EDSS was evaluated at the latest follow-up after ≥24 months of therapy. Six patients were excluded from EDSS-based analyses due to missing disability assessments. A complete-case analysis approach was applied, as missingness was attributable to incomplete clinical documentation rather than disease-related factors.

Relapses occurring within the first six months were included, recognizing delayed therapeutic onset. To preserve anonymity and patient confidentiality, no identifiable information was collected. All participants provided written informed consent prior to data collection.

### 2.3. Outcome Measures

The primary outcome was the ARR, defined as the total number of relapses during the treatment period divided by the number of years of follow-up. A relapse was operationalized as the appearance of new or worsening neurological symptoms persisting for at least 24 h in the absence of fever or infection, followed by partial or complete recovery, consistent with established clinical definitions [19]. Secondary outcomes included EDSS score categories (mild: 0–3.5; moderate: 4.0–5.5; severe: ≥6.0) and the incidence and number of treatment-related adverse effects.

### 2.4. Statistical Analysis

Normality of continuous variables was assessed prior to analysis using visual inspection of histograms and distribution characteristics. Variables showing approximate normal distribution were analyzed using independent samples *t*-tests, whereas non-normally distributed variables were summarized using medians and ranges, and non-parametric approaches were considered where appropriate. Categorical variables were analyzed using chi-square test or Fisher’s exact test for categorical variables. All tests were two-tailed, with a significance threshold set at *p* ≤ 0.05. Statistical analyses were performed using IBM SPSS Statistics for Windows, Version 29 (IBM Corp., Armonk, NY, USA).

## 3. Results

### 3.1. Participant Characteristics

A total of 122 patients with relapsing–remitting multiple sclerosis (RRMS) were included in the analysis. Of these, 87 patients (71.3%) were receiving fingolimod, 33 patients (27.0%) were treated with IFN-β1a, and 2 patients (1.6%) were being treated with other therapies. These two patients were included only in descriptive analyses and were excluded from all comparative outcome analyses. Table 1 summarizes the sociodemographic and clinical characteristics of the study population. Most patients in the cohort were females (73.8%), with a male-to-female ratio of approximately 1:2.8. This sex distribution is consistent with the known epidemiology of relapsing–remitting multiple sclerosis in Jordan and other Middle Eastern populations, where female predominance is well documented [20]. The median age at disease onset was 27 years (range: 14–54 years), and the largest age group was between 31 and 40 years (40.2%). The majority of participants were nonsmokers (67.2%), and over half (53.3%) had attained a college-level education or higher. Most patients were unemployed (58.2%) at the time of assessment, and 54.1% were married. Initial presenting symptoms varied; sensory or pain-related symptoms were most common (48.4%), followed by visual disturbances (35.2%), dizziness or imbalance (8.2%), motor deficits (4.9%), and other symptoms (3.3%). A family history of MS was reported by 15.5% of participants.

### 3.2. Disability Assessment

**Figure 1:**
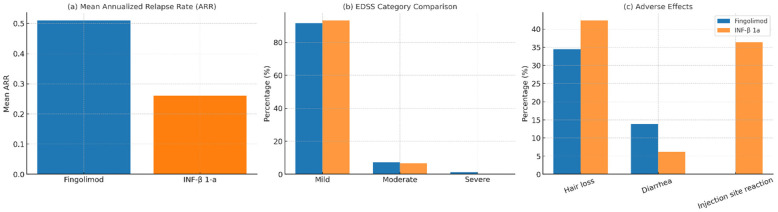
Comparison of key clinical outcomes and adverse effects between treatment groups. (**a**) Mean annualized relapse rate (ARR) in patients treated with fingolimod and interferon beta-1a. (**b**) Distribution of Expanded Disability Status Scale (EDSS) categories at last follow-up. (**c**) Frequency of reported adverse effects by treatment group.

Disability status was systematically evaluated using EDSS. Among 114 patients with available EDSS data, the majority (92.2%) had mild disability (EDSS ≤ 3.5), while 6.9% had moderate disability (EDSS 4.0–5.5), and 0.9% had severe disability (EDSS ≥ 6.0) (Table 1), (Figure 1). Table 2 summarizes the correlation between current medications and patient outcomes. There were no statistically significant differences in disability severity between the fingolimod and IFN-β1a groups (*p* = 1.00). In the fingolimod group, 77 patients had mild, 6 had moderate, and 1 had severe disability. In the IFN-β1a group, 26 patients had mild and 2 had moderate disability, with none classified as severe (Table 2). Overall, the absence of statistically significant differences in EDSS categories between treatment groups reflects similar observed disability distributions at the time of assessment, rather than equivalence in long-term disability outcomes.

### 3.3. Relapse Rate Comparison

**Figure 2:**
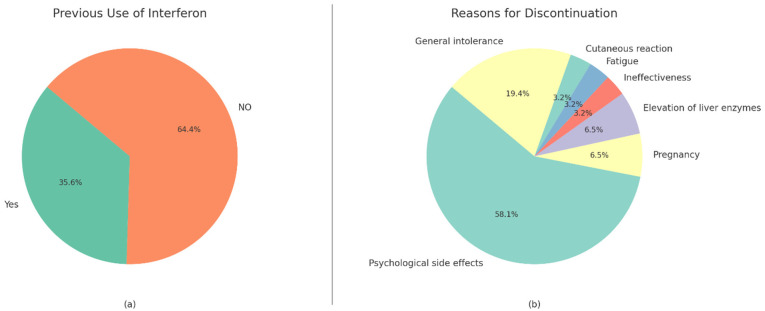
Interferon use history and reasons for discontinuation among fingolimod-treated patients. (**a**) Proportion of patients who had previously received interferon therapy prior to switching to fingolimod. (**b**) Reported reasons for interferon discontinuation among patients with prior interferon exposure.

Next, we measured the risk for relapse and compared the effect of both drugs. The mean ARR across the cohort was 0.44. Patients treated with fingolimod had a higher observed ARR compared to those receiving IFN-β1a (0.51 vs. 0.26; *p* = 0.016), with a mean difference of 0.245 (95% CI: 0.047–0.442) (Table 2) (Figure 2). This difference corresponded to a moderate effect size (Cohen’s *d* ≈ 0.49; *r* = 0.24). This difference reflects observed outcomes in a real-world cohort and should be interpreted in the context of treatment sequencing and baseline disease characteristics.

### 3.4. Symptom Burden

Symptom analysis revealed no statistically significant differences between the treatment groups across four major domains: visual, bowel/bladder, pyramidal, and sensory symptoms (all had *p* > 0.05). Visual symptoms were reported by 61.7% of patients on fingolimod and 60.6% of those on IFN-β1a. Pyramidal symptoms, including weakness and balance issues, were present in 66.7% of fingolimod patients and 69.7% of IFN-β1a patients. Similarly, bowel/bladder and sensory symptoms were evenly distributed across groups (Table 2).

### 3.5. Adverse Effects and Treatment Switching

The incidence and number of adverse drug reactions differed significantly between groups. Patients receiving IFN-β1a reported a frequent injection site reaction (36.4%), which is absent in the fingolimod group (*p* < 0.001). Other adverse effects, such as hair loss (reported in 36.7% of the overall cohort) and diarrhea (11.7%), did not significantly differ between groups. The mean number of reported side effects was higher in the IFN-β1a group (0.82 ± 0.63) compared with the fingolimod group (0.49 ± 0.58; *p* = 0.024). (Table 2) (Figure 1). Of the patients currently on fingolimod, 31 patients (35.6%) had previously received IFN-β1a (Figure 2). The most frequently reported reason for switching was psychological side effects (64.5%) most commonly including anxiety, depressive symptoms, mood disturbances, and emotional distress related to long-term injectable therapy, followed by general intolerance (19.4%), pregnancy (6.5%), elevation of liver enzymes (6.5%), fatigue (3.2%), ineffectiveness (3.2%), and cutaneous reactions (3.2%) (Figure 2).

## 4. Discussion

This study provides real-world evidence comparing the clinical outcomes of fingolimod and IFN-β1a in the management of RRMS in a Jordanian cohort. The findings demonstrate a significantly higher ARR among patients treated with fingolimod, whereas no significant difference was observed in disability progression, as assessed by EDSS. Both treatments exhibited distinct safety profiles, with the exception of frequent injection site reactions with IFN-β1a.

The direction of association observed in this study differs from that reported randomized controlled trials. In the TRANSFORMS study, fingolimod demonstrated superior relapse reduction compared with IFN-β1a, a benefit that was maintained in the TRANSFORMS extension under controlled trial conditions [21,22]. Similarly, in pediatric relapsing–remitting multiple sclerosis, the PARADIGMS trial showed greater relapse reduction with fingolimod versus IFN-β1a [23]. The discrepancy between these trial results and the present real-world findings is consistent with clinical practice in the Middle East, where fingolimod is frequently prescribed as second-line or escalation therapy for patients with higher disease activity or inadequate response to injectable treatments. Consequently, relapses observed in this setting likely reflect treatment sequencing and underlying disease severity rather than pharmacologic efficacy. In the large FINOMENA multicentered cohort from the MENA region, patients initiated on fingolimod typically exhibited higher disease activity and greater prior DMT exposure, which contributed to variability in ARR outcomes despite overall treatment efficacy [24]. Similar patterns were reported in a Lebanese cohort, where fingolimod showed favorable clinical and radiological benefits but was predominantly used in patients who had previously failed injectable therapies [25]. The most important methodological consideration in interpreting these findings is confounding by indication. In routine clinical practice in Jordan, fingolimod is predominantly prescribed as escalation therapy for patients with higher inflammatory disease activity or inadequate response to injectable treatments. In our cohort, more than one-third of fingolimod-treated patients had prior exposure to IFN-β1a, strongly suggesting systematic baseline differences between treatment groups that could not be fully captured in retrospective data. The higher observed relapse rates among fingolimod-treated patients therefore likely reflect underlying disease severity and treatment sequencing rather than reduced pharmacologic efficacy.

Population-specific biological factors may also influence treatment response. Jordan and the broader Middle East report a high prevalence of vitamin D deficiency and variable genetic susceptibility profiles which modulate relapse risk and treatment effectiveness [6,7]. A recent Jordanian study investigating cytokine gene expression found that fingolimod significantly downregulated pro-inflammatory cytokines and improved MRI lesion burden, supporting its biological effectiveness in local populations despite variability in clinical relapse outcomes [26]. Additionally, prior studies have shown that interferon formulations and fingolimod differentially modulate inflammatory mediators in RRMS, further supporting the biological plausibility of treatment effects even when observational relapse measures are influenced by confounding [27]. Collectively, these findings suggest that the elevated annualized relapse rate observed among fingolimod-treated patients in our cohort more likely reflects underlying disease characteristics and treatment sequencing rather than reduced pharmacologic efficacy.

The lack of observed differences in EDSS scores should not be interpreted as equivalence between treatments. Regarding the absence of significant differences in EDSS scores between treatment groups, EDSS progression over short- to mid-term follow-up is often minimal, even when relapse rates differ [15]. EDSS assessed at a single time point has limited sensitivity to early or subtle disability progression and does not capture early functional decline, cognitive symptoms, and fatigue, which may be better assessed using composite measures incorporating MRI, relapse activity, and patient-reported outcomes. Regional studies have similarly demonstrated that clinical disability progression in RRMS often lags behind inflammatory activity, reinforcing the importance of multimodal monitoring strategies [24].

Regarding safety, IFN-β1a was associated with significantly more injection site reactions, consistent with its well-known tolerability profile [12,13]. In contrast, fingolimod appeared better tolerated in this cohort, aligning with evidence from regional and international cohorts [24,25]. However, the potential for serious adverse effects (including bradycardia, macular edema, and infection risk) were not systematically assessed in this study and therefore cannot be excluded [16].

Adherence-related and socioeconomic factors play a critical role in shaping real-world treatment outcomes in Jordan. A notable observation in this study is that more than one-third of patients receiving fingolimod had previously been treated with IFN-β1a, with psychological and tolerability-related adverse effects being the most common reasons for switching. This aligns with Jordanian data showing that adherence to DMTs remains suboptimal, with socioeconomic hardship, injection fatigue, and limited patient education contributing to nonadherence [28]. These factors are particularly important in Jordan, where MS predominantly affects young adults navigating employment, academic responsibilities, and family life. Therefore, treatment decisions should integrate patient preference, mental health considerations, and lifestyle needs in addition to clinical factors.

From both clinical and health-system perspectives, these findings highlight the importance of real-world evidence for guiding multiple sclerosis management in resource-constrained settings such as Jordan, where disease-modifying therapies constitute a major component of MS-related healthcare expenditure and access to newer high-efficacy treatments remains limited [29]. The higher observed relapse rates among fingolimod-treated patients are most plausibly explained by treatment escalation in individuals with more active disease rather than reduced therapeutic efficacy, underscoring the need for early disease activity assessment and timely treatment sequencing. Differences in tolerability profiles further emphasize the importance of standardized safety monitoring and structured adherence support programs, particularly in public hospitals where injectable therapies remain widely used.

Pharmacoeconomic studies indicate that the relative cost-effectiveness of fingolimod and IFN-β1a is highly sensitive to drug pricing, loss of exclusivity, payer perspective, and healthcare system structure, with fingolimod appearing cost-effective under certain willingness-to-pay thresholds following price reductions, although results vary across settings [30,31]. In Jordan’s public healthcare sector—where medication availability, monitoring capacity, and patient out-of-pocket costs differ from high-income settings—real-world data such as those presented here are essential for pragmatic treatment selection and resource planning. Strengthening multidisciplinary MS care services and aligning therapeutic choices with system-level constraints may help improve outcomes, reduce relapse-related hospitalizations, and inform context-specific national treatment strategies.

In summary, these findings contribute valuable region-specific evidence on the comparative effectiveness and tolerability of fingolimod and IFN-β1a in Jordanian RRMS patients. When interpreted alongside existing Jordanian and Middle Eastern studies, the results highlight the complex interplay between biological, behavioral, and systemic factors that shape treatment outcomes. Future prospective, multicenter studies incorporating MRI metrics, adherence assessment, and longitudinal disability tracking are warranted to further refine treatment strategies for MS in Jordan and the wider region.

The study is limited by its retrospective design, single-center sample, and lack of MRI and adherence data, which may restrict the generalizability of the findings. Nevertheless, it highlights critical gaps in regional MS management, including the need for multicenter registries, standardized data collection, and broader access to newer therapies.

By contextualizing treatment outcomes within the realities of the Jordanian healthcare system, this work contributes region-specific insight and underscores the importance of individualized, patient-centered therapeutic strategies. Future prospective studies integrating imaging and adherence metrics are warranted to validate and extend these findings.

## 5. Conclusions

In this real-world cohort from Jordan, observed differences in ARRs between fingolimod- and IFN-β1a-treated patients were strongly influenced by treatment sequencing and baseline disease activity and should not be interpreted as comparative effectiveness estimates. Although IFN-β1a was associated with a lower observed relapse rate, disability distributions measured by the EDSS did not differ at the assessment time point, reflecting the limitations of retrospective, cross-sectional evaluation. These findings emphasize the importance of individualized treatment selection in routine practice, with IFN-β1a remaining a suitable first-line option for patients with milder inflammatory activity who can tolerate injectable therapy, while fingolimod is commonly reserved for escalation in patients with more active disease, prior injectable intolerance, or when oral administration may improve adherence, provided that appropriate safety monitoring is available. Prospective multicenter studies incorporating standardized MRI measures and longitudinal disability assessment are needed to better inform treatment sequencing strategies in Jordan.

## Figures and Tables

**Table 1:** Sociodemographic and clinical characteristics of patients.

Variable	Number (%)
**Total**	122 (100)
**Age**	
<20	4 (3.3)
21–30	32 (26.2)
31–40	49 (40.2)
41–50	26 (21.3)
>50	11 (9)
**Age at onset**	
Median (Range)	27 (14–54)
**Gender**	
Male	32 (26.2)
Female	90 (73.8)
**Smoking**	
Current or former smoker	40 (32.7%)
Never smoker	82 (67.2%)
**Level of education**	
College or higher	65 (53.3)
High school	44 (36.1)
Less than high school	13 (10.6)
**Employment status**	
Employed	51 (41.8)
Unemployed	71 (58.2)
**Marital status**	
Single	56(45.9)
Married	66(54.1)
**Initial presentation**	
Visual	43 (35.2)
Motor	6 (4.9)
Sensory/pain	59 (48.4)
Dizziness/imbalance	10 (8.2)
Others	4 (3.3)
Disability (EDSS) *	
0–3.5	107 (92.2)
4–5.5	8 (6.9)
6–10	1 (0.9)
**Current medication**	
Fingolimod	87 (71.3)
Interferon	33 (27)
Others	2 (1.6)
**ARR**	
Mean (CI)	0.44 (0.33–0.55)
**Family history of MS**	
Yes	19 (15.5)
No	103 (84.5)

* Six patients were excluded due to the absence of EDSS.

**Table 2:** The correlation between current medications and patient outcomes.

Variable	Number (%)	Fingolimod	INF-β 1-a	*p* Value
**Total**	120 (100)	87 (72.5)	33 (27.5)	
EDDS *	114 (100)			1
Mild	105 (92.1)	77 (91.7)	28 (93.3)	
Moderate	8 (7)	6 (7.1)	2 (6.7)	
Severe	1(0.9)	1 (1.2)	0 (0)	
**ARR**				**0.016**
Mean	0.44	0.51	0.26	
**Visual symptoms**				0.150
0	46 (38.3)	33 (37.9)	13 (39.4)	
1–2	38 (31.7)	24 (27.6)	14 (42.4)	
>2	36 (30.0)	30(34.5)	6 (18.2)	
**Bowel/bladder symptoms**				0.322
0	68 (56.7)	52 (59.8)	16 (48.5)	
1–2	38 (31.7)	24 (27.6)	14 (42.4)	
>2	14 (11.7)	11 (12.6)	3 (9.1)	
**Pyramidal symptoms**				0.592
0	39 (32.5)	29 (33.3)	10 (30.3)	
1–2	32 (26.7)	21 (24.1)	11 (33.3)	
>2	49 (40.8)	37 (42.5)	12 (36.4)	
**Sensory symptoms**				0.742
0	28 (23.3)	19 (21.8)	9 (27.3)	
1–2	50 (41.7)	36 (41.4)	14 (42.4)	
>2	42 (35.0)	32 (36.8)	10 (30.3)	
**Sexual symptoms**				0.281
Yes	10 (8.3)	9 (10.3)	1 (3.0)	
No	110 (91.7)	78 (89.7)	32 (97.0)	
Number of side effects (0–2) **				**0.024**
Mean ± SD	0.58 ± 0.60	0.49 ± 0.58	0.82 ± 0.63	
**Hair loss**				0.420
Yes	44 (36.7)	30 (34.5)	14 (42.4)	
No	76 (63.7)	57 (65.5)	19 (57.6)	
**Diarrhea**				0.345
Yes	14 (11.7)	12 (13.8)	2 (6.1)	
No	106 (88.3)	75 (86.2)	31 (93.9)	
**Injection site reaction**				**0.000**
Yes	12 (10)	0 (0)	12 (36.4)	
No	108 (90)	87 (100)	21 (63.6)	

* Six patients were excluded due to the absence of EDSS. ** The range ‘(0–2)’ under number of side effects indicates the observed minimum and maximum count per patient. Two patients receiving other therapies were excluded from this analysis (*n* = 120); 2-sided *p*-value. A *p*-value ≤ 0.05 is considered significant (bold).

## Data Availability

The data utilized and analyzed during the current study are available from the corresponding author upon request.

## Abbreviations

MS—Multiple Sclerosis; RRMS—Relapsing-Remitting Multiple Sclerosis; DMT—Disease-Modifying Therapy; IFN-β1a—Interferon beta-1a; ARR—Annualized Relapse Rate; EDSS—Expanded Disability Status Scale.
